# Transcriptional changes during crown-root development and emergence in barley (*Hordeum vulgare* L.)

**DOI:** 10.1186/s12870-024-05160-y

**Published:** 2024-05-22

**Authors:** Dieu Thu Nguyen, Filip Zavadil Kokáš, Mathieu Gonin, Jérémy Lavarenne, Myriam Colin, Pascal Gantet, Véronique Bergougnoux

**Affiliations:** 1 Czech Advanced Technology and Research Institute, Palacký University Olomouc, Olomouc, Czechia; 2 Department of Biochemistry, Faculty of Science, Palacký University Olomouc, Olomouc, Czechia; 3 UMR DIADE, Université de Montpellier, IRD, CIRAD, Montpellier, France; 4 Present address: Masaryk Memorial Cancer Institute, Brno, Czechia

**Keywords:** Barley (*Hordeum vulgare* L.), Crown roots, Transcriptome, Emergence

## Abstract

**Background:**

Roots play an important role during plant growth and development, ensuring water and nutrient uptake. Understanding the mechanisms regulating their initiation and development opens doors towards root system architecture engineering.

**Results:**

Here, we investigated by RNA-seq analysis the changes in gene expression in the barley stem base of 1 day-after-germination (DAG) and 10DAG seedlings when crown roots are formed. We identified 2,333 genes whose expression was lower in the stem base of 10DAG seedlings compared to 1DAG seedlings. Those genes were mostly related to basal cellular activity such as cell cycle organization, protein biosynthesis, chromatin organization, cytoskeleton organization or nucleotide metabolism. In opposite, 2,932 genes showed up-regulation in the stem base of 10DAG seedlings compared to 1DAG seedlings, and their function was related to phytohormone action, solute transport, redox homeostasis, protein modification, secondary metabolism. Our results highlighted genes that are likely involved in the different steps of crown root formation from initiation to primordia differentiation and emergence, and revealed the activation of different hormonal pathways during this process.

**Conclusions:**

This whole transcriptomic study is the first study aiming at understanding the molecular mechanisms controlling crown root development in barley. The results shed light on crown root emergence that is likely associated with a strong cell wall modification, death of the cells covering the crown root primordium, and the production of defense molecules that might prevent pathogen infection at the site of root emergence.

**Supplementary Information:**

The online version contains supplementary material available at 10.1186/s12870-024-05160-y.

## Background

Despite their hidden nature, roots play fundamental roles in plant growth and development, ensuring not only anchorage in the soil, but also water and nutrient up-take, and interaction with soil microorganisms which contribute to the regulation of crop productivity [1]. Plants are able to dramatically modify their root system in response to environmental and nutrient conditions [2–4]. Even though studying root system in natural environment is laborious, in situ root phenotyping methods have evolved from invasive and destructive methods, such as “shovelomics”, to non-destructive and non-invasive 2-D and 3-D methods [5]. Those methods allowed to visualize the foraging activity of roots and contributed to understand the molecular mechanisms regulating root growth and development. The identification of genetic determinants of root development offers the opportunity to manipulate root system architecture with the aim to improve crop productivity.

Cereals represent the most important source of proteins in human diet, with wheat, rice, maize representing in 2020 more than 35% of proteins consumed per capita each day, far before poultry and pig meat (11.8%; FAO stat). However, cereal production suffers from climatic changes [6]. Climatic changes are characterized by increasing number and severity of episodes of drought and heat that are responsible for drops in yield [7]. As roots are the first interface between plant and edaphic environment, sensing changes in soil moisture and nutrient availability, they represent a good target for new breeding programs.

The typical root system of cereals is composed of the embryonically formed primary and seminal roots, and of the post-embryonically formed adventitious roots that are shoot-borne roots, called crown roots (CR). CR appear quickly after germination at the junction between the stem and the root. Primary, seminal and crown roots have the ability to form lateral roots of different order. All together, they form the characteristic fibrous root system of monocots [8]. In some monocots, embryonic primary and seminal roots are not persisting, and the mature root system is constituted exclusively from the CR [9, 10].

Maize (*Zea mays* L.), wheat (*Triticum aestivum* L.), rice (*Oryza sativa* L.) and barley (*Hordeum vulgare* L.) represent 39% of the crops cultivated worldwide. Due to its significance for human feeding and the relatively low size of its genome, rice has been chosen since 1985 as the model plant for monocots. This was also justified by the fact that the cultivated rice has been adapted to many culture conditions and a wide range of genetically and phenotypically diverse varieties are available. Its genome has been sequenced and several mutant collections have been created [11]. The development of rice functional genomics, QTL analysis and genome-wide association studies allowed to decipher the molecular mechanisms controlling the initiation and development of CR [12, 13]. However, the tropical growth habit of rice is different from that of cereals grown under the European temperate climate, and one could address whether molecular mechanisms controlling root development in rice are conserved among temperate-type cereals.

Barley is the fourth most cultivated cereals worldwide (http://faostat.fao.org/). Except of human food supply, barley is used in brewery, distilling industry and animal feeding. Its area of production is very large, covering a range of extreme environments, suggesting an ability to adapt to different culture conditions [14], and consequently an under-exploited genetic diversity with high potential for new breeding programs [15]. The development of high-throughput sequencing in the last decade resulted in the availability of barley genome and its annotation [16–19]. Finally, the genetic modification of barley is possible since *Agrobacterium*-mediated transformation can be successfully achieved both from immature embryo [20] or embryogenic pollen culture [21]. All these criteria, together with its diploid genome, make that barley can serve as a complementary model in addition to rice to study the root system development in cereals. In the present study, we present data from the whole transcriptome analysis of barley seedlings stem base developing CR and discuss the possible mechanisms underlying CR development and emergence in barley.

## Material and methods

### Material and cultivation conditions

The spring barley cultivar Golden Promise (cv. GP) was used in this study. For the purpose of the experiment, grains were immersed for 30 s in 70% ethanol, washed 3 times in water, then surface sterilized in 3% sodium hypochlorite for 5 min and extensively rinsed in water. Finally, grains were placed on wet filter paper in Petri dishes and placed in the dark for 2 days at 4°C. Germination was induced by transferring petri dishes to a culture chamber with a long-photoperiod (16h-day/8h-night; 400 µmol.s^−2^.m^−1^) and a temperature regime of 21°C-day/18°C-night. The day of appearance of the primary roots and coleoptile through the seed coat was considered as the germination day. One day after germination (1DAG), half of the germinated young seedlings were sampled, separating roots, stem bases (1 mm fragment from the root junction) and shoots; the rest of the seedlings were kept for 3 more days on wet filter paper in petri dishes, then transferred into a mini-hydropony system containing a modified half-strength Hoagland solution [22]. At 10 days after germination (10DAG), seedlings were harvested, separating roots, stem bases (1,5–2 mm fragment from the root junction)) and shoots. Each sample (roots, stem bases and shoots) represented a pool of 5 seedlings; samples were immediately flash frozen in liquid nitrogen and stored in a deep-freezer until use. The experiment was conducted into three independent biological replicates.

### Imaging of crown root primordia by light and confocal microscopy

For light microscopy, plantlets were grown as previously described. The developing young seedling was separated from the rest of the grain; primary roots were removed from the aerial part with a blade, and a 2 mm-long section from the stem base was excised with a scalpel and immediately immersed in fixative (4% paraformaldehyde in 1X PBS, pH 7.4). Vacuum was applied twice for 1 min 30 s. Fixative was changed, and samples were placed overnight at 4°C in the dark. The next day, samples were washed 4 times in 1X PBS for 15 min. Samples were dehydrated in an ethanol series from 50 to 100%. Samples were embedded in resin (Technovit® 7100; Electron Microscopy Sciences) according the instructions. Sections of 4.5 µm were obtained with a HYRAX M40 microtome (Zeiss), stained with either periodic acid Schiff-naphtol blue black (PAS-NBB) or lugol, and mounted in Clearium® Mounting Media (Electron Microscopy Sciences). Sections were observed with an Imager M2 microscope equipped with an Axio Cam MrC5 camera (Zeiss). Pictures were acquired and analyzed with the ZEN software (Zeiss).

The imaging of the stem base by confocal microscopy was performed as previously described [23]. The 1 mm-long and 2 mm-long stem base of barley seedlings were harvested at 1DAG and 10DAG, respectively, and immediately immersed in ½ Hoagland solution at 4°C overnight. They were embedded in 5% agarose in sterile distilled water (w/v). Agarose blocks were mounted into the Automate 880 (Phiv platform, MRI imaging facility, Montpellier, France, https://www.mri.cnrs.fr/), a custom machine combining an LSM NLO 880 multiphoton microscope (Zeiss, https://www.zeiss.com) equipped with a Chameleon Ultra II tunable pulsed laser (690–1080 nm range excitation; Coherent, https://www.coherent.com) and an HM 650V vibratome (Microm Microtech, http://www.mm-france.fr) allowing automation of sample cutting (60 µm) and instant imaging. The images were obtained with a 20x/1.0NA (2.4 mm WD) objective. The instrument was controlled using ZEN and the custom package ZEN EXTENSION NECE (Zeiss). Images were acquired through the whole stem base. The imaged sections were aligned with Fiji, an application of ImageJ [24]. For 3D reconstruction, aligned sections were used as input in IMARIS9.1 (Imaris, https://imaris.oxinst.com).

### Evans blue staining

Cell death at the site of root emergence was observed by Evans blue staining as described previously [25]. Briefly, 10-day-old seedlings of cv. GP grown in hydroponic conditions were immersed in 2% (w/v) Evans blue, prepared in water, for 3 min and subsequently washed in water. Evans blue penetrates only dead cells. Samples were observed with a Zeiss Axio Zoom v16 Stereo Microscope equipped with a camera. Serial pictures were taken and analyzed with ZEN software (Zeiss).

### Library preparation and transcriptomic analysis

For both 1DAG and 10DAG, seedlings were grown as described. Grain tegument and the rest of the endosperm were removed to keep only the young seedling composed of the small primary and seminal roots and the stem enclosed in the coleoptile. The stem base (1 to 2 mm-long), corresponding to the zone of emergence of CR in barley, was excised, flash frozen in liquid nitrogen and stored in deep freezer at -80°C until they were used for RNA extraction and transcriptomic analyses.

Total RNA was extracted according the instructions of the ZymoResearch Plant RNA extraction kit and treated by TURBO™ DNase. Libraries were prepared from 2.5 µg of total RNA according to the instructions of the Illumina TruSeq1 Stranded mRNA Sample Preparation Kit (Illumina). Library concentration was assessed with the Kapa Library Quantification Kit (Kapa Biosystem) and all libraries were pooled to the final 8 pM concentration before cluster generation and sequencing. The clusters were generated using an Illumina TruSeq1 PE Cluster Kit v3cBot HS and sequenced on HiSeq PE Flow Cell v3 with a HiSeq 2500 Sequencing System. Three independent biological replicates were sequenced for each sample.

The paired-end reads (50bp-long) generated by sequencing were quality checked and trimmed prior mapping to the reference genome of barley cv. Morex IBSC_v1 [26] using the Tophat2 splice-read mapper with default parameters [27]. The mapped reads were quantified using HTSeq with respect to the stranded library [28]. The differentially expressed gene expression was calculated with the DESeq2 package [29]. Only genes harboring a |log2foldchange|≥ 1 and *p*_adjusted value_ ≤ 0.05 were considered to be significantly up-or down-regulated, and further analyzed. The gene functional annotation and enrichment was done using the MapMan BIN ontology in Mercator4 [30, 31]. Indeed, the widely used Gene Ontology (GO) comprises more than 34,000 terms organized in 3 categories: “Biological process”, “Molecular Function” and “Cellular component”. This rich annotation can lead to a strong redundancy and problem to visualize data from RNAseq. In opposite, Mapman/Mercator was specifically developed for plants with the goal to visualize omics data, such as transcriptome, on plant pathways [32]. Currently, MapMan ontology covers 27 functional top-categories, referred to as BIN [31, 33]. In parallel, the GO enrichment analysis limited to “Biological process” was performed with ShinyGO [34]: the 40 most enriched GO terms were sorted based on their fold enrichment. All data are available at the NCBI archive database under the GEO accession number GSE87737 (https://www.ncbi.nlm.nih.gov/geo/query/acc.cgi?acc=GSE87737).

### Analysis of gene expression by quantitative real-time PCR (qPCR)

To confirm the differential expression of several interesting genes, qPCR analysis was performed with i) samples used for RNA-seq focused on the stem base region of seedlings at 1DAG or 10DAG, and ii) with samples obtained from various tissues (roots, stem bases and shoots) of cv. GP seedlings at 1DAG and 10DAG.

For the analysis of gene expression in different tissues, total RNA was extracted from roots, stem bases and shoots. Each sample was prepared in 3 independent biological replicates, with each replicate being composed of tissues of 5 seedlings. For this purpose, seedlings were grown and harvested as described for the preparation of the RNAseq libraries. Total RNA was extracted with the Quick-RNA Plant Kit (Zymo Research) following manufacturer’s instructions. Any potential trace of genomic DNA was removed with an additional treatment with 2U of TURBO™ DNase (ThermoFisher scientific) for 30 min at 37°C, and subsequent precipitation with lithium chloride. Total RNA was finally dissolved in RNAse-free water. The concentration was determined with a NanoDrop™ One/OneC Microvolume UV–Vis Spectrophotometer (ThermoFisher scientific).

For both experiments, the oligo(dT)_18_-based cDNA synthesis was performed from 2 µg of the total RNA according the instructions of the RevertAid First Strand cDNA synthesis kit (Thermo Fisher Scientific). For real-time PCR, cDNA samples were diluted 10 times and used in a reaction containing 1X gbSG PCR master mix (Generi biotech), 200 nM of each primer and 500 nM of ROX as a passive reference. Three technical replicates were run for each sample on a StepONE Plus thermocycler (Applied Biosystems) in a two-step amplification program. The initial denaturation at 94°C for 2 min was followed by 45 cycles of 94°C for 5 s and 60°C for 20 s. A dissociation curve was obtained for each sample. To determine the best reference gene(s), we both used the information from the available literature, our previous research work, but also the advance of the RefGenes tool under Genevestigator [35]. The expression profile across the different samples was analyzed for 6 potential reference genes (data not shown). Their stability across samples was determined using RefFinder [36] that integrates the currently available major computational programs (geNorm, Normfinder, BestKeeper, and the comparative ΔCt method) to compare and rank the tested candidate reference genes. Based on our data, *ACT* (AK248432), *Hv5439* (AK360511) and *EIF5A2* (AK357300; [37]) genes were found to be the most stable across the samples under investigation and were consequently used as normalizer. Cycle threshold values for the gene of interest were normalized in respect to the three reference genes and the geometric mean of expression was calculated. The relative expression was determined using the ΔΔCt mathematical model corrected for the PCR efficiency (E) [38]. The relative quantification was estimated in comparison with either “crown-1DAG_RNAseq_” or “roots-1DAG” sample. Primers were designed using Primer3Plus (https://primer3plus.com/cgi-bin/dev/primer3plus.cgi). Their specificity was checked by blast restricted to barley genome, validated by the dissociation curve and sequencing of the amplified product. Sequences of primers are given in Supplemental table S1.

### Prediction of putative *cis*-regulating elements related to ethylene and cell death

The presence of putative *cis*-regulating elements related to ethylene was predicted with PlantPAN3.0 [39] for 3 genes with a potential involvement in cell death during CR emergence. For this purpose, the 1 kb-long sequence upstream of the ATG was retrieved from barley genome. The sequences were scanned with the “Promoter analysis” tools using the TF binding motifs database from rice.

### Statistical analysis

The GraphPad Prism version 9.2.0. was used to prepare all figures and evaluate the statistical analysis. In all cases, the statistical significance was assessed by a two-way ANOVA followed by a Bonferroni multiple comparisons test.

## Results and discussion

### Crown-root primordia development in the stem base of the spring barley cv. Golden Promise

**Fig. 1:**
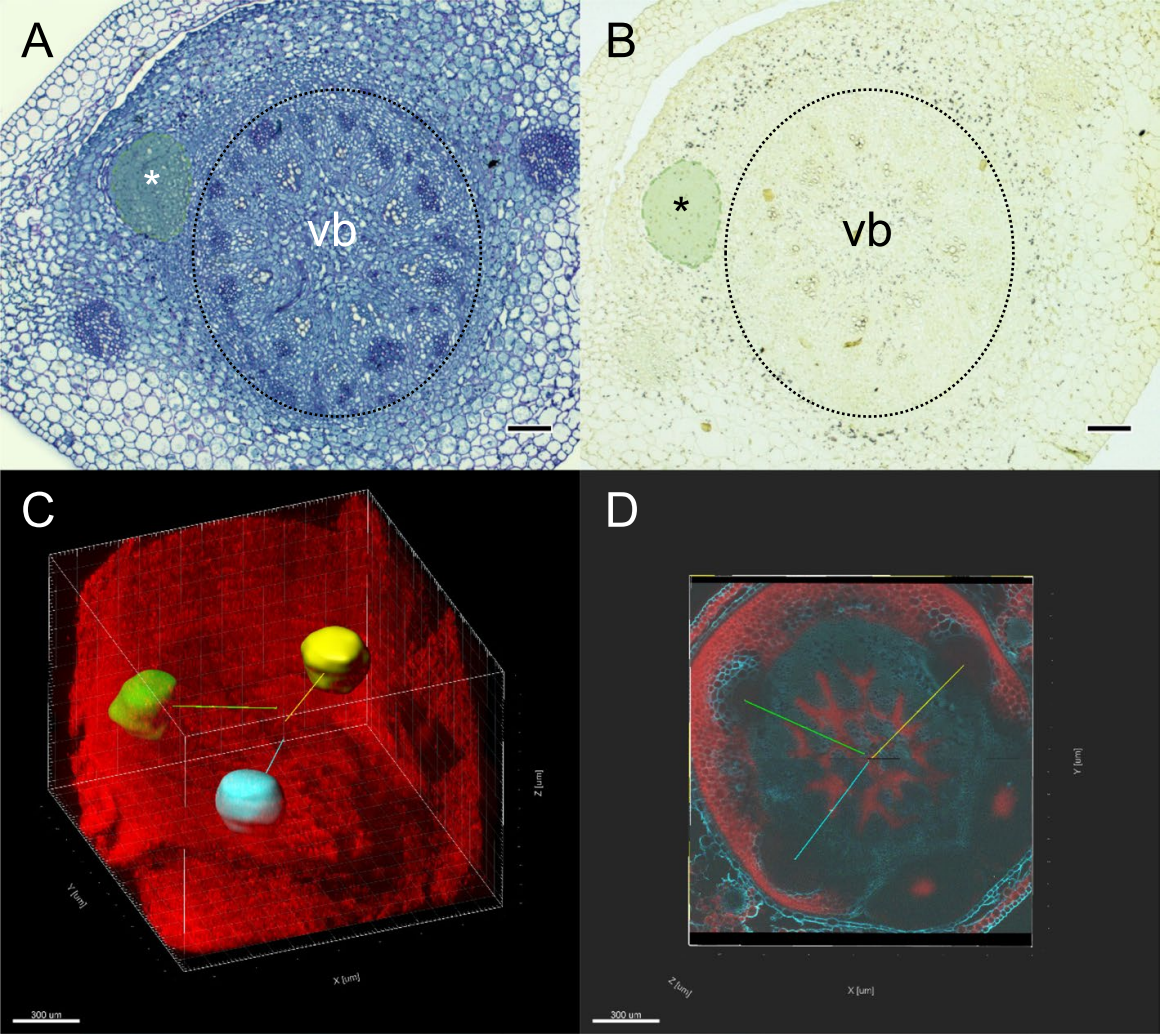
Crown-root primordia development in young barley seedlings. In 3DAG-old seedling, one primordium (*) is formed at the outermost side of the pericycle (**A**; PAS-NBB staining). The pericycle is surrounded by cells rich in starch as shown by the presence of dark dots after staining with Lugol solution (**B**). In (A) and (B), the dashed circle indicates the limit of the vascular bundles (vb). **C** Result of a block reconstruction in the 3D transparency mode of the whole stem base of a 10 DAG-old barley seedling. CR primordia are identified by different color. **D** z-axis cross section; the colored traits indicate the angle between each CR primordia. Bars in A and B represent 100 µm

In rice, CR originate from the ground meristem cells adjacent to the peripheral cylinder of vascular bundles in the stem [40]. This meristematic cell layer can be assimilated to a shoot pericycle-like tissue that is surrounded by a starch-rich endodermis [41, 42]. Using classical histology and biphoton confocal microscopy, we evidenced that in barley the first CR is already formed at 3DAG (Fig. 1A) at the outermost side of the pericycle, that is characterized by cells rich in starch (Fig. 1B). We designed this region as ground meristem in comparison to rice. The earliness of CR primordia during seedling establishment has already been described in other monocots. Indeed, in rice, CR emerge from the coleoptilar node already 2 to 3 DAG [43] and 10 DAG in maize [44]. The study of 10 DAG-old seedlings by biphoton confocal microscopy suggested that CR primordia are formed sequentially, i.e. one after each other, with a seemingly averaged distance of 76 µm and 140° angle between two CR primordia (Fig. 1C and D). This disagrees with the observation done in rice or maize, where several CR are produced in whorls [23]. Therefore, further anatomical study will be required to describe in details the formation of CR in barley. In the stem base of 1DAG-old seedlings no CR initium could be observed by biphoton confocal microscopy, neither by classical histology (data not shown).

For the purpose of the study, we compared the transcriptome of the stem base of 1DAG-old seedlings when no CR primordia could be seen to that of 10 DAG-old seedlings when 1 to 3 CR already emerged, forming a “bud” at the surface of the stem base.

### Transcriptomic changes in the stem base of 10DAG seedlings of spring barley and functional annotation of differential expressed genes (DEGs)

Gene profiling of the CR development in barley was investigated by RNA-seq whole transcriptome analysis, comparing the transcriptome of stem base of 10DAG seedlings to that of 1DAG seedlings of the spring barley cv. GP. For this purpose, 6 libraries (GP-1DAG-rep1, GP-1DAG-rep2, GP-1DAG-rep3, GP-10DAG-rep1, GP-10DAG-rep2 and GP-10DAG-rep3) were constructed from the basal portion of barley seedling’s stem. These libraries were sequenced using an Illumina HiSeq2000 system. After adaptor trimming and masking low-complexity or low-quality sequence, we obtained 54–77 million raw single-end reads. The 45 bp-reads were mapped to the reference genome of barley cv. Morex IBSC_v1 [26] using TopHat with default parameters. The averaged total single mapping rates of all samples was around 92.3% (Supplemental table S2). Differentially expressed genes were determined with DESeq2. Taking the limits of *p*_adjusted value_ < 0.05 and a log2foldchange excluding values from -1 to 1, there were 5,264 DEGs between GP-10DAG and GP-1DAG, of which 2,932 were up-regulated (UP10DAG, Supplemental table S3) and 2,333 were down-regulated (DO10DAG, Supplemental table S4) in the stem base of 10DAG-seedlings compared to the stem base of 1DAG-seedlings.

**Fig. 2:**
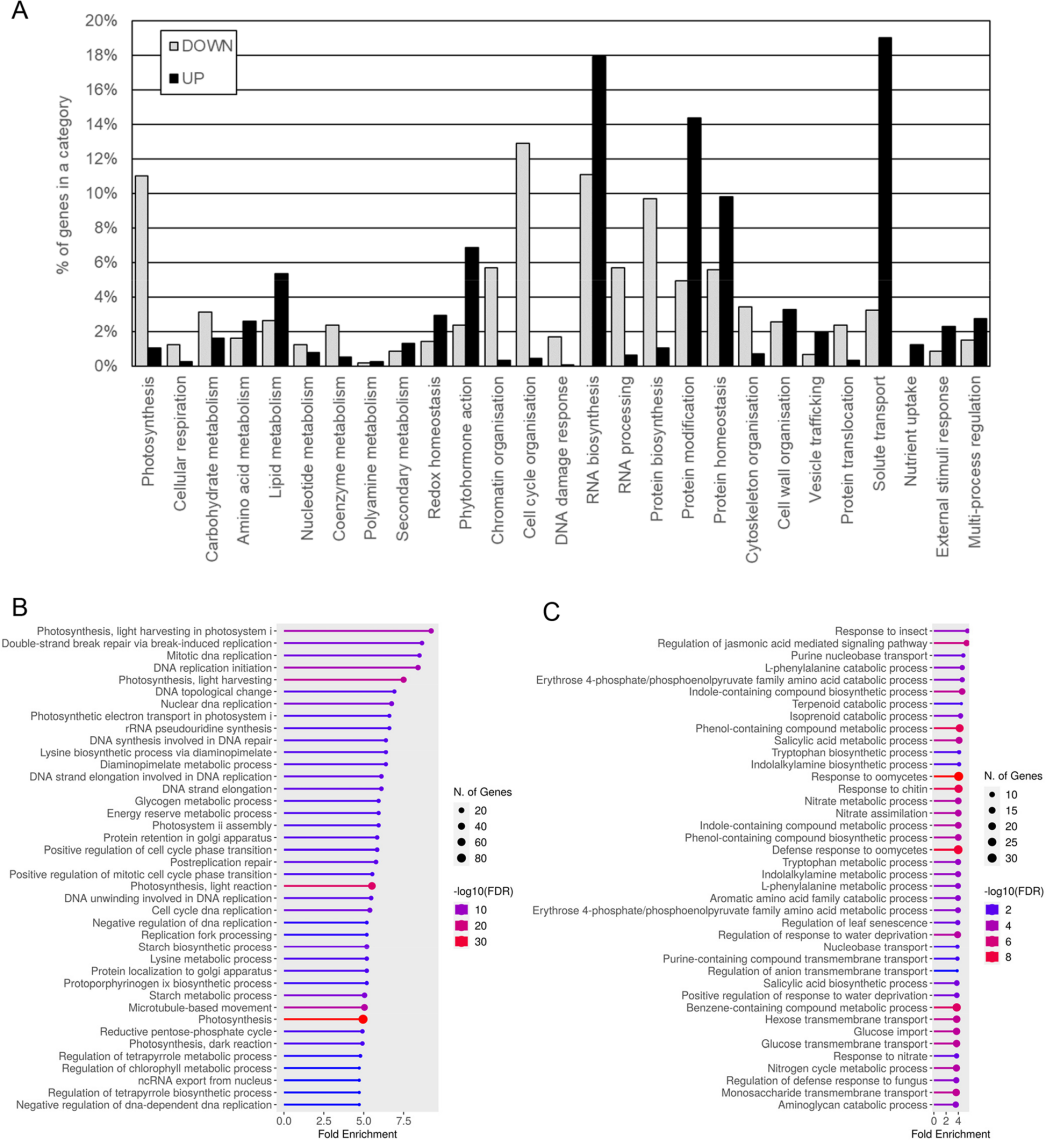
**A** Functional annotation of genes differentially regulated in the stem base of 10 DAG-old seedling of barley. The functional annotation was done with Mercator [30, 31]; the number of genes in one category is expressed as a percentage of the total number of genes that were annotated. Gene Ontology (GO) enrichment of genes down-regulated (**B**) and up-regulated (**C**) in the stem base of 10DAG-old seedlings of barley. The analysis was performed with ShinyGO [34]

The MapMan gene functional annotation of the barley reference transcriptome was done in Mercator4 [31]: among the 24,210 predicted genes, only 11,289 genes were annotated and categorized into different BINS. The functional annotation of the DEG revealed that different pathways are represented in the stem bases of 1DAG and 10DAG seedlings (Fig. 2A). Indeed, among genes up-regulated in the stem base of 1DAG seedlings, 35.4% of the genes were related to cell cycle organization, chromatin organization, RNA biosynthesis or RNA processing, and 20.2% were related to protein (biosynthesis, homeostasis or modification), suggesting that profound molecular modifications occurred in the stem base of seedlings that will enter the program of CR initiation and development. This was supported by the annotation enrichment analyses (Table 1 and Fig. 2B).

**Table 1:** Functional annotation enrichment of genes differentially regulated in the crown of 10 DAG-old seedling of barley cv. Golden Promise. The automated annotation was performed using the Mercator resource [30]. The enrichment analysis was done with Mercator4 v6.0 that uses a one-sided Fisher’s exact test to performe the over-represenation analysis

**MapMan4 category number**	**Context of protein function**	**#Genes of interest IN MapMan4 category**	**#Genes of interest NOT IN MapMan4 category**	**#Background genes IN MapMan4 category**	**#Background genes NOT IN MapMan4 category**	**Enrichment factor**	***p*-value**	**FDR-adjusted*p*-value**
**BIN functional enrichment of UP-regulated genes**								
10	Redox homeostasis	21	2,910	58	24,152	2.99	< 0.01	0.0180
26	External stimuli response	31	2,900	97	24,113	2.64	< 0.01	< 0.01
24	Solute transport	225	2,706	940	23,270	1.98	< 0.01	< 0.01
11	Phytohormone action	100	2,831	440	23,770	1.88	< 0.01	< 0.01
15	RNA biosynthesis	198	2,733	1,015	23,195	1.61	< 0.01	< 0.01
18	Protein modification	155	2,776	893	23,317	1.43	< 0.01	< 0.01
**BIN functional enrichment of DOWN-regulated genes**								
19	Protein homeostasis	8	2,325	10	24,200	8.3	< 0.01	0.0126
15	RNA biosynthesis	20	2,313	34	24,176	6.1	< 0.01	< 0.01
1	Photosynthesis	121	2,212	237	23,973	5.3	< 0.01	< 0.01
3	Carbohydrate metabolism	17	2,316	38	24,172	4.64	< 0.01	< 0.01
13	Cell division	148	2,185	423	23,787	3.63	< 0.01	< 0.01
16	RNA processing	42	2,291	135	24,075	3.23	< 0.01	< 0.01
17	Protein biosynthesis	107	2,226	492	23,718	2.26	< 0.01	< 0.01
20	Cytoskeleton organisation	48	2,285	261	23,949	1.91	< 0.01	0.0135
12	Chromatin organisation	66	2,267	378	23,832	1.81	< 0.01	< 0.01

The genes up-regulated in the stem base of 10DAG seedlings belonged to categories: solute transport, (19.0%), RNA biosynthesis (17.9%), protein modification and homeostasis (24.2%), phytohormone action (6.9%), lipid metabolism (5.4%), cell wall organization (3.3%), redox homeostasis (2.9%). Supported by the annotation enrichment analyses (Fig. 2C and Table 1), our data suggested that a cellular/tissular organization occurs in the stem base of 10DAG seedlings.

**Fig. 3:**
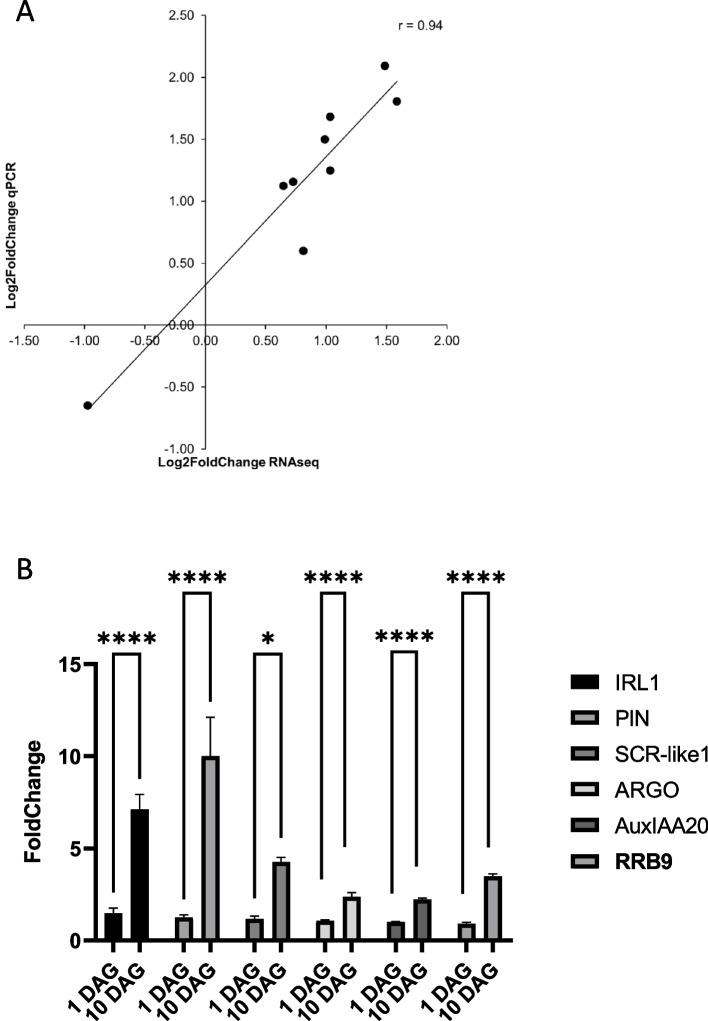
**A** Comparison of expression as determined by RNA-seq and real-time PCR. All expression data were normalized to the log2 scale. The coefficient of Pearson correlation was determined to be *r* = 0.94. **B** Validation of differential expression by qPCR of 6 genes with a potential role in CR initiation and development. qPCR was performed on the same samples as those used for RNAseq analysis. Normalization was done using 3 most stable reference genes: *Actin*, *Hv5439* and *EIF152*. The graph shows means ± SEM (*n* = 3). The statistical significance was assessed by a two-way ANOVA followed by a Bonferroni multiple comparisons test (GraphPad Prism 9.2.0). ****: adjusted *P*-value < 0.0001; *: adjusted *P*-value < 0.005

**Fig. 4:**
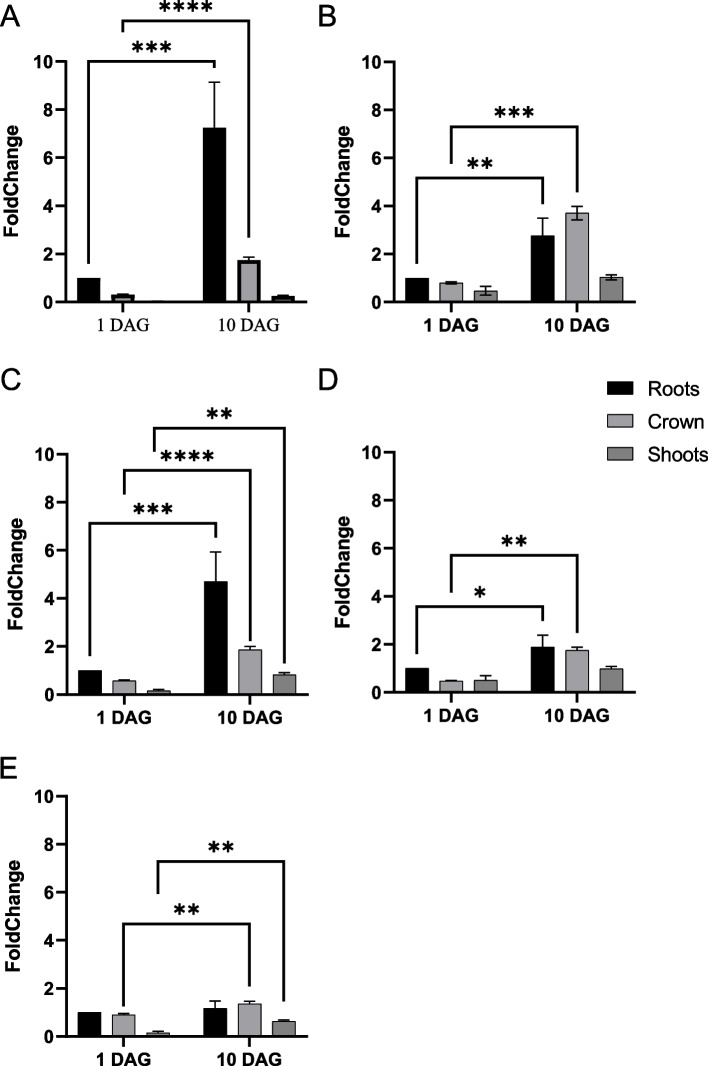
Gene expression analysis by qPCR of PIN (**A**), SCR-like1 (**B**), ARGO (**C**), AuxIAA20 (**D**) and RRB9 (**E**) in the roots, stem base and shoots of cv. Golden Promise seedlings grown for 10 days in hydroponic conditions. Normalization was done using 3 reference genes: *Actin*, *Hv5439* and *EIF152*. The graph shows means ± SEM (*n* = 3). The statistical significance was assessed by a two-way ANOVA followed by a Bonferroni multiple comparisons test (GraphPad Prism 9.2.0). ****: adjusted *P*-value < 0.0001; ***: adjusted *P*-value < 0.001; **: adjusted *P*-value < 0.001; *: adjusted *P*-value < 0.005

Changes in gene expression determined by RNAseq were validated by qRT-PCR analysis. For this purpose, the change in expression of 9 genes was investigated (Fig. 3A) and the correlation between RNA-seq data analysis and qPCR was confirmed (coefficient of Pearson correlation, *r* = 0.94; Fig. 3A). Five genes with a potential role in CR development (*PIN-FORMED-LIKES/PILS*, *SCARECROW-LIKE1* /*SCR-like1*, *ARGONAUTE/ARGO*, *AuxIAA20* and *RESPONSE REGULATOR9*/*RRB9*) were confirmed by qRT-PCR to be up-regulated in the stem base of 10DAG seedlings (Fig. 3B). Their expression was also significantly increased in the primary and seminal roots of the 10DAG seedlings (Fig. 4). Even though our study did not focus on the lateral root development, these genes could be also involved in the initiation and development of lateral roots. Indeed, it has been demonstrated that development of lateral roots and CR shared common molecular regulators [8, 13].

### Cell identity priming and cell cycle activation during crown-root development in barley

In the stem base of 1DAG-seedlings, 12 genes encoding cyclin, cyclin-dependent kinases were up-regulated, suggesting an important activation of the cell cycle. In addition, Cell Division Control (CDC) proteins and anaphase promoting proteins were also up-regulated suggesting that active cell division occured. We also found that 32 kinesin and kinesin-related proteins were up-regulated in the stem base of 1DAG seedling initiating CR. Kinesins form a superfamily of microtubule motor proteins and trigger the unidirectional transport of vesicles and organelles, affect microtubule organization and cellulose microfibril order. They were also described to be involved in cell division and growth [45].

In Arabidopsis, lateral root initiation is characterized by founder cell identity priming, cell cycle activation and asymmetric division of the founder cells. Auxin maxima are responsible for the up-regulation of cyclins and cyclin-dependent kinases (CDKs) and the concomitant repression of CDK repressors such as the Kip-related proteins (KRP1 and 2) which inhibits the G1/S transition [46, 47]. Interestingly, in Arabidopsis, auxin alone is not sufficient for lateral root initiation. Moreno-Risueno and coworkers demonstrated the existence of a so-called “oscillation zone” which is primordial for the spatial and temporal definition of lateral root initiation sites [48]. Transcription factors of the ARF (auxin response factor), NAC (NAM-No Apical Meristem, ATAF1-Arabidopsis thaliana Activation Factor 1, and CUC2-Cup-shaped Cotyledon), myeloblastosis (MYB) and SOMBRERO families are important for the determination of lateral root initiation sites [48].

### Hormonal status during crown root development in barley

Genes belonging to the category “hormone metabolism” accounted for a large proportion of sequences over-represented in the stem base of 10DAG seedlings (7%), suggesting an important modification in hormonal status during the development and emergence of CR in barley.

Auxin is probably the most important hormone that regulates initiation of CR. Among the genes abundant in the stem base of 10DAG seedlings, we found 27 genes related to auxin (IAA) metabolism, signal transduction or induced by auxin. IAA maxima are fundamental for root primordia formation and emergence [49, 50]. In rice, OsPIN1 plays a role during CR development rather than during the CR primordia initiation. Other PINs (OsPIN2, OsPIN5b and OsPIN9) are likely to be involved in this process [50]. In our data, two auxin transporter-like proteins and one auxin efflux carrier were identified as up-regulated in the stem base of 10DAG seedlings. These 3 auxin transporters might participate in establishing auxin gradient required for CR development in barley. The release of free auxin from conjugates is often neglected. In the present study, we identified a gene annotated as *IAA-amino acid hydrolase* (*ILR1*). IAA-amino acid conjugates function in both the permanent inactivation and temporary storage of auxin, participating thus in auxin homeostasis regulation. ILR allows releasing free IAA from the amino acid conjugates [51]. In Arabidopsis, the triple hydrolase mutant, *ilr1 iar3 ill2*, developed fewer lateral roots than the wild-type, demonstrating the importance of IAA release from conjugate in the initiation of lateral roots [52]. Whether IAA is released from IAA-conjugates via ILR1 to support CR primordia formation and development in barley represents an interesting challenge to solve.

Our study revealed that at least 18 genes related to ethylene metabolism and signaling pathway were up-regulated in the crown of 10DAG seedling, when CR are already initiated and are emerging from the stem. In rice, ethylene induces the death of epidermal cells at the site of CR emergence. Thus through the crack of the epidermis the newly formed root can emerge without damages [25]. Our data suggest that in barley, the emergence of CR is possibly correlated with death of cortex and epidermal cell in an ethylene-mediated response. This is supported by the fact that a gene associated with development and cell death was also up-regulated in the stem base of 10DAG seedling.

Abscisic acid (ABA) is another important hormone. Its role as inhibitor or stimulator of plant growth and development is a constant question of debate [53]. ABA has a dual role in root development: whereas it stimulates the initiation and primordia formation in different species, it often inhibits the emergence from the stem and the subsequent elongation of the root [54]. Fifteen genes related to ABA synthesis and signaling pathway were found to be up-regulated in the stem base of 10DAG seedlings. Among these genes belonged two transcripts annotated as 9-cis-epoxycarotenoid dioxygenase (NCED) which catalyzes the first committed step of ABA synthesis. This suggested that ABA synthesis takes place in the stem base of seedlings developing CR and that ABA is also important for the development of CR in barley. Nevertheless, deeper studies would be necessary to precisely determine the role of ABA in the different steps of CR development, i.e. primordium initiation, emergence and root elongation.

We also found that a gene encoding a gibberellin 2-oxidase, highly abundant in the crown of barley seedling, was up-regulated in 10DAG-seedlings. GAox2 are responsible for the degradation of active gibberellins (GAs). In poplar, GAs negatively regulate lateral root, specifically inhibiting root primordium initiation. The role of GA2ox in regulating GAs homeostasis was also proved in the same study [55]. In rice, overexpressing *GA2ox* led to the decrease of endogenous GAs and enhanced CR root growth [56]. Similarly, silencing of *SLENDER1*, a negative regulator of the GA signaling pathway, resulted in lower number of CR in rice [57]. It is thus tempting to postulate that GAs are inhibitors of CR initiation and development in barley and a precise regulation of its homeostasis via GA2ox is required.

### Emergence of CR induces cell death and cell wall modification

The molecular events of lateral root and CR emergence are still unclear. Nevertheless, two different modes of root emergence have been assumed: i) “active removal” of the cortical cells most probably induced by root primordia, implying death of cells covering the root primordia, and ii) “mechanical breakage” of epidermal cells by the tension resulting from the growth of the root [58].

**Fig. 5:**
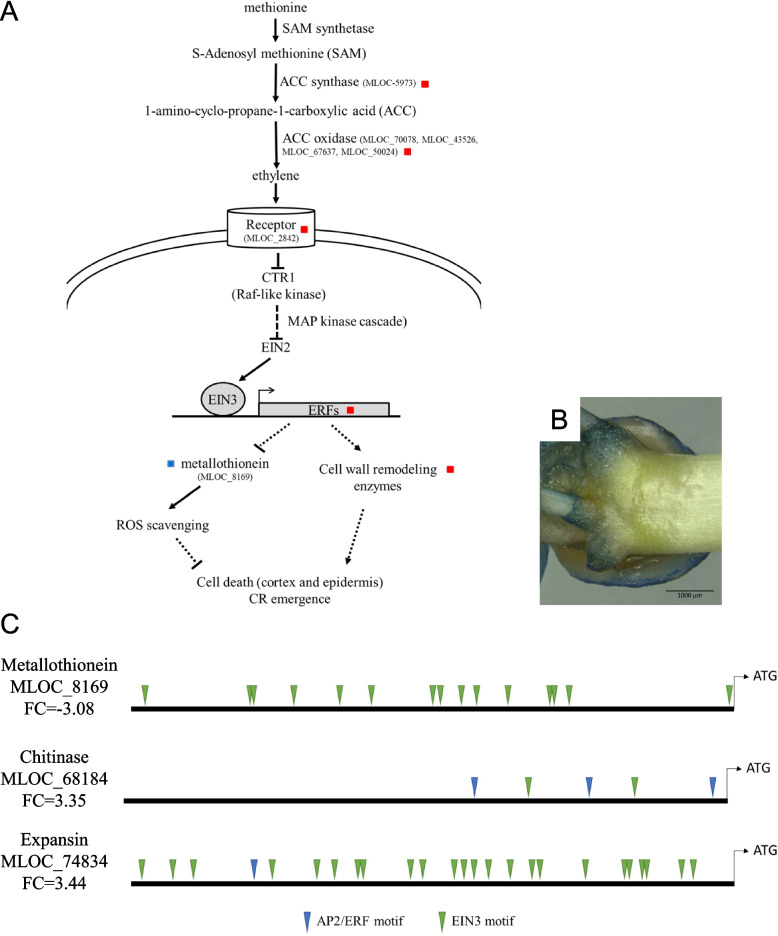
Involvement of ethylene in cell death during crown root emergence in barley cv. GP. **A** Ethylene biosynthetic and signaling pathway in the context of cell death. Genes identified in the RNAseq data as differentially expressed are indicated (MLOC); colored scares indicate whether they were up-regulated (red) or down-regulated (blue) in the stem base of 10 days-old seedlings. **B** Evan's blue staining indicates the cell death of the epidermal cell at the site of crown root emergence. **C** Prediction of the presence of ethylene-related *cis-*regulating elements (AP2/ERF and EIN3 motifs). The prediction was done with PlantPAN3.0, using the rice database.

In rice, the emergence of CR happens concomitantly to death of nodal epidermal cells above CR primordia (Mergemann and Sauter, 2000). In maize, Park and coworker reported the formation of a cavity in the cortex of primary root around the lateral root primordia, resulting probably from the death of the cells [59]. Apoptosis of epidermal cells is controlled by ethylene and is mediated by reactive oxygen species (ROS), which are also involved in CR primordia growth [60], allowing coordinating CR growth with local weakening of the epidermal cell barrier [61]. In the present study, we demonstrated that genes involved in the ethylene pathway were up-regulated in the stem base of 10DAG seedlings (Fig. 5A). The use of the Blue Evan’s staining showed that cell death occurred at the site of emergence of CR (Fig. 5B). It was shown that epidermal cells covering CR primordia might be targeted to die, as they contain lower amount of the METALLOTHIONEIN2b (MT2b), a scavenger of ROS [61]. Interestingly, a gene encoding a metallothionein was strongly down-regulated in the stem base of 10 DAG-old seedlings, suggesting a reduction in ROS scavenging. Our transcriptomic data suggest that emergence of CR in barley is correlated with cell death, mediated by ethylene and ROS. Thirteen genes annotated as (endo)chitinases were up-regulated in the stem base of 10 DAG-old seedlings. Chitinases are glycosyl hydrolases that catalyze the degradation of chitin, a major constituent of fungi cell wall and exoskeleton of insects. Commonly induced upon pathogen attack, they were for long associated with plant defense. However, growing evidence determined that (endo)-chitinases have different function during plant growth and development. The analysis of Arabidopsis chitinases revealed 5 classes, some of them having functions in “cell wall synthesis” or in “cell rescue, defense, cell death and aging” [62]. The role of class IV chitinase in cell death was recently reported in pepper [63]. Genes of the functional category “cell wall” were overrepresented among genes up-regulated in the stem base of 10 DAG-old seedling (13%), indicating that profound modifications occur when CR primordia form and develop. These genes are mainly related to pectin lyases, expansins and xyloglucan endotransglucosylases/hydrolases (XEGs). In Arabidopsis, the newly formed lateral root has to pass through 3 cell layers: endodermis, cortex and epidermis [64]. Cells are particularly well attached to each other, especially epidermal cells. Genes encoding proteins affecting cell wall-property integrity (expansins, pectin lyases or XEGs) are expressed in tissues covering the emerging lateral root primordia. The activity of these enzymes most probably promotes cell separation in advance of developing lateral root primordia to avoid damages of the root meristem [65]. Moreover, the cell-wall properties could contribute to the number of lateral root produced [66]. Indeed, the high-affinity auxin importer LIKE AUX1 (LAX3) is an important regulator of lateral root emergence. Its expression in cells situated over the lateral root primordia regulates the activity of cell wall remodeling enzymes, which are likely to promote cell separation in advance of developing lateral root primordia [66]. In the present study, a gene encoding LAX3, was up-regulated in the crown of 10 DAG-old seedlings where CR are formed and emerging.

The analysis of the 1.5 kb promoter sequence of three genes encoding a metallothionein, a chitinase and an expansin revealed the presence of numerous AP2/ERF and EIN3 motifs, reinforcing the hypothesis that those genes could be regulated by ethylene during crown root emergence (Fig. 5C).

It was supposed that the site of epidermis breakage in relation to root emergence may represent a site of infection by pathogens [58]. Benzoxazinoids are plant secondary metabolites involved in resistance against pathogens. They are present in grasses and in some dicots. Maize was the most important source of studies concerning these compounds. Their biosynthesis branches off from tryptophan at the indole-3-glycerol phosphate, which is converted into indole by indole-3-glycerol phosphate lyase (BX1). Four cytochrome P450 monooxygenase (BX2-BX5) are responsible for the subsequent production of DIBOA, the most effective benzoxazinoid [67]. In maize, benzoxazinoids defense molecules were found to accumulate at the site of CR emergence, preventing pathogenic infections which could occur during the crack of the epidermis. It is interesting to observe that in the stem base of barley 10 DAG-old seedling, genes encoding enzymes of the benzoxazinoid biosynthesis were up-regulated. Two genes were orthologues to the maize *indole-3-glycerol phosphate lyase* (*BX1*) and others were related to *BX2* and *BX4*, suggesting that this synthetic pathway was stimulated during CR development and emergence.

## Conclusion

We analyzed the transcript profiling of barley young seedlings to increase our understanding on the mechanisms regulating CR development and emergence in this species. Our study constitutes the first step toward understanding the molecular and physiological mechanisms involved in development and emergence of CR in barley, and represents an important resource on barley for the scientific community working on CR formation. Whereas we identified similar role of auxin, CK and others hormones in the process as described in other species, our study brings novelty concerning the last stage of CR development, i.e. emergence. Further functional studies of the identified genes will be required to characterize their involvement in CR formation.

### Supplementary Information

Supplementary Material 1.

## Data Availability

The sequenced raw reads generated in this study have been submitted to the National Center for Biotechnology Information (NCBI) with BioProject ID: GSE87737 (https://www.ncbi.nlm.nih.gov/geo/query/acc.cgi?acc=GSE87737). The two-raw spring barley cultivar Golden Promise was grown and multiplied in the glasshouses of CATRIN, Palacky University in Olomouc. The original stock was obtained from Genbank IPK-Gaterlesben, Germany. The methods involved in this study were carried out in compliance with local and national regulations.
